# AN INTEGRATIVE ANALYSIS OF STUDENT AND INSTRUCTOR PERSPECTIVES ON DISCUSSING SUICIDE IN THE CLASSROOM

**DOI:** 10.1093/geroni/igae098.0883

**Published:** 2024-12-31

**Authors:** Raven Weaver, Autumn Decker, Ashley Robillard, Maksim Vasylenko, Madison Kidner, Cory Bolkan

**Affiliations:** Washington State University, Pullman, Washington, United States; Pacific University, Hillsboro, Oregon, United States; Washington State University, Pullman, Washington, United States; Washington State University, Pullman, Washington, United States; Washington State University, Pullman, Washington, United States; Washington State University, Pullman, Washington, United States

## Abstract

Suicide is often considered a triggering topic and may pose challenges in classroom settings. Suicide is a lifespan issue with older adults having the highest risk and it being a leading cause of death among young adults. To improve both personal response and professional readiness, it is important for college students to learn about suicide in the classroom. This integrative, cross-sectional study relied on a quantitative survey of college students (n= 313; Mean age:19.5; 71% Women) on effective ways for learning about and discussing sensitive topics, like suicide, as well as qualitative semi-structured interviews of college faculty teaching death and dying courses (n = 27, 83% women; 94% White), who shared their perspectives on teaching about sensitive topics including death, dying, and suicide. Most students wanted to learn more about suicide (74%) and about available resources to support individuals at risk of suicide (86%). Conversely, content analysis of faculty perspectives revealed they typically avoided discussing suicide in the classroom; not all faculty felt prepared or comfortable having suicide-based discussions and many feared that discussions would exacerbate students’ depression, anxiety, or suicide ideation. We highlight the importance of collaborative methods and co-designing curricula to deliver trauma-informed, evidence-based instruction. There is a need for more integration of campus and external resources, and the development of EBPI trainings around suicide prevention on college campuses, as well as a need to prepare future gerontological professionals to be able to detect and support older adults in mental health crises.

